# Assessing the prognostic role of androgen receptor expression in non-metastatic triple-negative breast cancer

**DOI:** 10.3389/fonc.2026.1785283

**Published:** 2026-03-23

**Authors:** Mahmoud Al-Masri, Yasmin Safi, Basim Aljalabneh, Isam Jarah, Hussam Ananzeh, Rama AlMasri, Osama Alayyan, Fade Alawneh, Mohammad Almasri

**Affiliations:** 1 Department of Surgery, King Hussein Cancer Center, Amman, Jordan; 2 Faculty of Medicine, The University of Jordan, Amman, Jordan; 3 Department of Medicine, King Hussein Cancer Center, Amman, Jordan

**Keywords:** androgen receptors, breast neoplasms, disease-free survival, prognosis, survival analysis, triple negative breast neoplasms

## Abstract

**Background:**

Triple negative breast cancer (TNBC) represents 15-20% of all invasive breast cancer with poor breast cancer survival. Androgen receptor (AR) expression in TNBC shows significant variability in the literature, with rates ranging from 7% to 75%. The association between AR expression and prognosis in TNBC remains controversial.

**Methods:**

A comparative retrospective design was utilized including all TNBC cases between 2014-2020. AR receptor expression was evaluated by immunohistochemical staining using whole tissue sections from archived paraffin-embedded formalin fixed blocks. Correlation of AR expression with standard clinical pathological factors and clinical outcomes of interest were assessed; including disease free survival (DFS), breast cancer specific survival (BCSS), and overall survival (OS).

**Results:**

149 patients with non-metastatic TNBC were included. 94 patients (63.3%) being AR negative and 55 patients (36.7%) were AR positive. No statistical difference in tumor characteristics between the two groups was found. The 5-year OS rates for AR negative and positive patients were 60% and 70% respectively (p= 0.021). However, this statistical significance was lost with longer follow-up (103 months, p=0.25). The 5-year DFS was similar for both groups (AR negative and AR positive, 64.3%, 62.9% respectively, p=0.39) in addition to the BCSS (73%, 78.8% respectively, p= 0.84). In univariable and multivariable analyses, AR expression did not significantly impact OS or DFS (HR: 0.71 and 1.07, p= 0.3, 0.8, respectively).

**Conclusion:**

In this study, AR status showed no association with DFS, BCSS, or OS; and was not a prognostic factor in TNBC. Further studies exploring the role of AR in TNBC are warranted as AR expression could be a potential target with antiandrogen therapy.

## 1 Introduction

Triple-negative breast cancer (TNBC) represents approximately 15–20% of all invasive breast cancer cases (1, 2). This subtype is characterized by its aggressive clinical behavior. It is associated with poorer outcomes, including reduced breast cancer-specific survival (BCSS) and overall survival (OS), particularly within the first three years following diagnosis. TNBC is also distinguished by a higher likelihood of recurrence and distant metastases compared to other breast cancer types (3). Given its aggressive nature and the limited availability of targeted therapeutic options, there is a pressing need to identify novel treatment strategies to enhance clinical outcomes for patients with TNBC.

Androgen receptors (AR), a class of steroid hormone receptors, are found to be overexpressed in approximately 70–90% of all breast cancer cases. However, in triple-negative breast cancer (TNBC), studies have reported a wide range in AR expression levels, with prevalence estimates varying from 7% to 75% (4–13).

The association between AR expression and its prognostic role in TNBC is contradictory, as some studies have reported a poorer outcome. In contrast, others showed favorable disease-free survival (DFS) and OS (14–17), in addition to some studies showing racial disparity with varied effects on prognosis (18).

Furthermore, numerous studies illustrate that AR expression is not associated with TNBC prognosis (19–30).

In this study, we present the clinicopathological features of our cohort of TNBC and determine AR expression and its use as a prognostic marker. To our knowledge, this is the first study that addresses this issue in the Levant area.

## 2 Methods

### 2.1 Study population and design

Data for this study were retrospectively collected from the cancer registry at the King Hussein Cancer Center for the period from 2014 to 2020. It involved individuals diagnosed with non-metastatic TNBC, defined by the lack of estrogen receptor (ER) and progesterone receptor (PR) expression (<1%) and HER2-negative status. Patients were further divided into two main groups according to AR expression: AR-positive and AR-negative. All receptor assessments, including ER, PR, HER2, and AR, were conducted on a single sample from the pre-treatment diagnostic biopsy.

For AR expression testing, tissues were fixed in 10% buffered formalin and embedded in paraffin. Immunohistochemical (IHC) staining for AR was performed using the Ventana Benchmark Ultra platform with a rabbit monoclonal antibody (clone SP107, Roche, Ventana Medical Systems Inc., Tucson, AZ), following the manufactures recommendations for this automated system. The recommended protocol that was used includes antigen retrieval using “Cell Conditioning 1” a Tris-based proprietary buffer with slightly basic pH for 64 minutes at 90 °C and primary antibody incubation for 32 minutes at 36 °C. The IHC staining was conducted on formalin-fixed, paraffin-embedded tissue sections and interpreted by experienced pathologists. Samples were considered positive if more than 1% of the cells showed staining, a threshold chosen based on the study conducted by Dubrava et al. in 2023 (31), which was informed by multiple reviewed literature sources.

The study compared survival outcomes, trends, and relationships between patients who tested positive for AR and those who tested negative using a comparative retrospective cohort methodology.

A comparative survival analysis between AR expression groups was the primary outcome. The time interval between diagnosis and the date of the last follow-up or the recurrence and metastasis of the disease was used to define DFS. Recurrence events included metastases to any other tissue or organ, ipsilateral breast, contralateral or ipsilateral axillae. The time from diagnosis to the date of the last follow-up or the date of death, regardless of cause of death, was defined as OS. The time interval between the date of diagnosis and the last follow-up date or the date of death directly attributable to breast cancer was known as BCSS.

### 2.2 Statistical analysis

R 4.2.1 was used to conduct the analysis. The descriptive analysis was displayed using percentages and frequencies. Cross-tabulations were used to show categorical data and Fisher’s exact or Chi-Square tests were used to evaluate associations. Regression models were built using variables that showed significance in univariate tests to do a Cox regression analysis later. A significant threshold of *p* < 0.05 was used. For the two cohorts, OS, DFS, and BCSS were estimated using the Kaplan-Meier method, and comparisons were made using the log-rank test.

## 3 Results

### 3.1 Patient and tumor characteristics

149 patients with non-metastatic TNBC were included in this study, with 94 patients (63.3%) being AR negative and 55 patients (36.7%) being AR positive. The median age was 49 years (19–84 years). 70 patients (47%) were post-menopausal and 79 (53%) were premenopausal. There were no statistical differences between the 2 groups in regard to BMI, family history of breast cancer and associated comorbidities. 78 patients (52.35%) were clinically staged as T2. Similarly, more than half (52.35%) of the cohort exhibited clinically positive lymph node metastasis. Grade III tumors comprised 75% of all patients. The majority of patients (97%) were stages II and I. Among the NACT group (n = 83), pCR was achieved in 9 patients (10.8%). Specifically, pCR occurred in 5 of 51 AR-negative patients (9.8%) and 4 of 32 AR-positive patients (12.5%). Non-pCR rates were 90.2% and 87.5% in the AR-negative and AR-positive groups, respectively. There was no statistically significant association between AR expression and pCR (p = 0.70). (**Table 1**).

**Table 1:** Comparison of clinicopathological variables by androgen receptor status.

Variable	Level	All (149)		Negative AR (94, 63.3%)		Positive AR (55, 36.7%)		P*
		N	%	N	%	N	%	
Age	Mean	49.05		47.88		51.07		0.412
	Median	49		47		51.5		
	SD	12.75323		13.33413		11.3984		
BMI	Mean	30.84		30.39		31.6		0.272
	Median	30		30		31		
	SD	6.533152		6.675402		6.204601		
Menopause Status	Postmenopausal	70	46.98%	40	42.55%	30	54.55%	0.157
	Premenopausal	79	53.02%	54	57.45%	25	45.45%	
Comorbidities	Yes	61	40.94%	43	45.74%	18	32.73%	0.119
	No	88	59.06%	51	54.26%	37	67.27%	
Family History of breast cancer	Yes	51	34.23%	28	29.79%	23	41.82%	0.51
	No	98	65.77%	66	70.21%	32	58.18%	
Clinical Stage	Ia	15	10.07%	10	10.64%	5	9.09%	0.412
	IIa	45	30.20%	27	28.72%	18	32.73%	
	IIb	47	31.54%	29	30.85%	18	32.73%	
	IIIa	32	21.48%	20	21.28%	12	21.82%	
	IIIb	7	4.70%	7	7.45%	0	0.00%	
	IIIc	3	2.01%	1	1.06%	2	3.64%	
Grade	I	2	1.34%	0	0.00%	2	3.64%	0.224
	II	28	18.79%	20	21.28%	8	14.55%	
	III	112	75.17%	70	74.47%	42	76.36%	
	NA	7	4.70%	4	4.26%	3	5.45%	
Clinical T Stage	T1	21	14.09%	14	14.89%	7	12.73%	0.156
	T2	78	52.35%	47	50.00%	31	56.36%	
	T3	43	28.86%	26	27.66%	17	30.91%	
	T4	7	4.70%	7	7.45%	0	0.00%	
Clinical N Stage	N0	71	47.65%	45	47.87%	26	47.27%	0.679
	N1	63	42.28%	41	43.62%	22	40.00%	
	N2 & N3	15	10.07%	8	8.51%	7	12.73%	
Neoadjuvant CTx	No	66	44.30%	43	45.74%	23	41.82%	0.585
	Yes	83	55.70%	51	54.26%	32	58.18%	
Neoadjuvant Chemotherapy (CTX) regimen	AC + Taxotere	71	85.54%	45	88.24%	26	81.25%	0.553
	AC	1	1.20%	0	0.00%	1	3.13%	
	Cyclophosphamide + Doxorubicin	2	2.41%	1	1.96%	1	3.13%	
	FEC + Paclitaxel	1	1.20%	1	1.96%	0	0.00%	
	Taxol	1	1.20%	1	1.96%	0	0.00%	
	NA	7	8.43%	3	5.88%	4	12.50%	
Clinical Response post Neoadjuvant	Complete	4	4.82%	3	5.88%	1	3.13%	0.936
	Partial	53	63.86%	32	62.75%	21	65.63%	
	Progressing	14	16.87%	8	15.69%	6	18.75%	
	No response	5	6.02%	3	5.88%	2	6.25%	
	NA	7	8.43%	5	9.80%	2	6.25%	
Pathological T Stage	T0	9	6.04%	6	6.38%	3	5.45%	0.668
	T1	50	33.56%	28	29.79%	22	40.00%	
	T2	28	18.79%	18	19.15%	10	18.18%	
	T3 & T4	59	39.60%	42	44.68%	17	23.64%	
	Tis	2	1.34%	0	0.00%	2	1.82%	
	Tx	1	0.67%	0	0.00%	1	1.82%	
Pathological N Stage	N0	73	48.99%	50	53.19%	23	40.00%	0.17
	N1	32	21.48%	22	23.40%	10	18.18%	
	N2	32	21.48%	14	14.89%	18	20.00%	
	Nx	12	8.05%	8	8.51%	4	7.27%	
For NACT group - Pathological Complete Response (pCR)	Non-pCR	74.0	89.2%	46.0	90.2%	28.0	87.5%	0.7
	pCR	9.0	10.8%	5.0	9.8%	4.0	12.5%	
Surgery Type	Breast Conservative surgery	68	45.64%	44	46.81%	24	43.64%	0.6
	Mastectomy with or without Reconstruction	81	54.36%	50	53.19%	31	40.00%	
Adjuvant CTx	No	68	45.64%	46	48.94%	22	40.00%	0.223
	Yes	81	54.36%	48	51.06%	33	60.00%	
Radiotherapy treatment	No	46	30.87%	31	32.98%	15	40.00%	0.467
	Yes	103	69.13%	63	67.02%	40	60.00%	
Non-surgical Treatment	Chemotherapy alone	46	30.87%	31	32.98%	15	27.27%	0.607
	Radiotherapy alone	7	4.70%	5	5.32%	2	3.64%	
	Chemoradiotherapy	96	64.43%	58	61.70%	38	69.09%	

*, *p*-value for mean age and BMI was calculated by t-test and all the other *p*-values were calculated by χ2 test.

### 3.2 Treatment modality

67 patients (45%) had breast-conserving surgery while 82 patients (55%) had mastectomy with or without reconstruction.

All patients received chemotherapy whether in the adjuvant or neoadjuvant setting. Approximately 70% of patients received adjuvant radiotherapy with no significant statistical difference in the modality of treatment between the two groups (**Table 1**).

### 3.3 Prognostic significance of AR expression

Using the reverse Kaplan–Meier method, the corrected median follow-up was 61.6 months (95% CI: 46.9–68.4). The numbers at risk at 60 months are provided in the **Supplementary Materials** (**Supplementary Table 1**).

**Figure 1:**
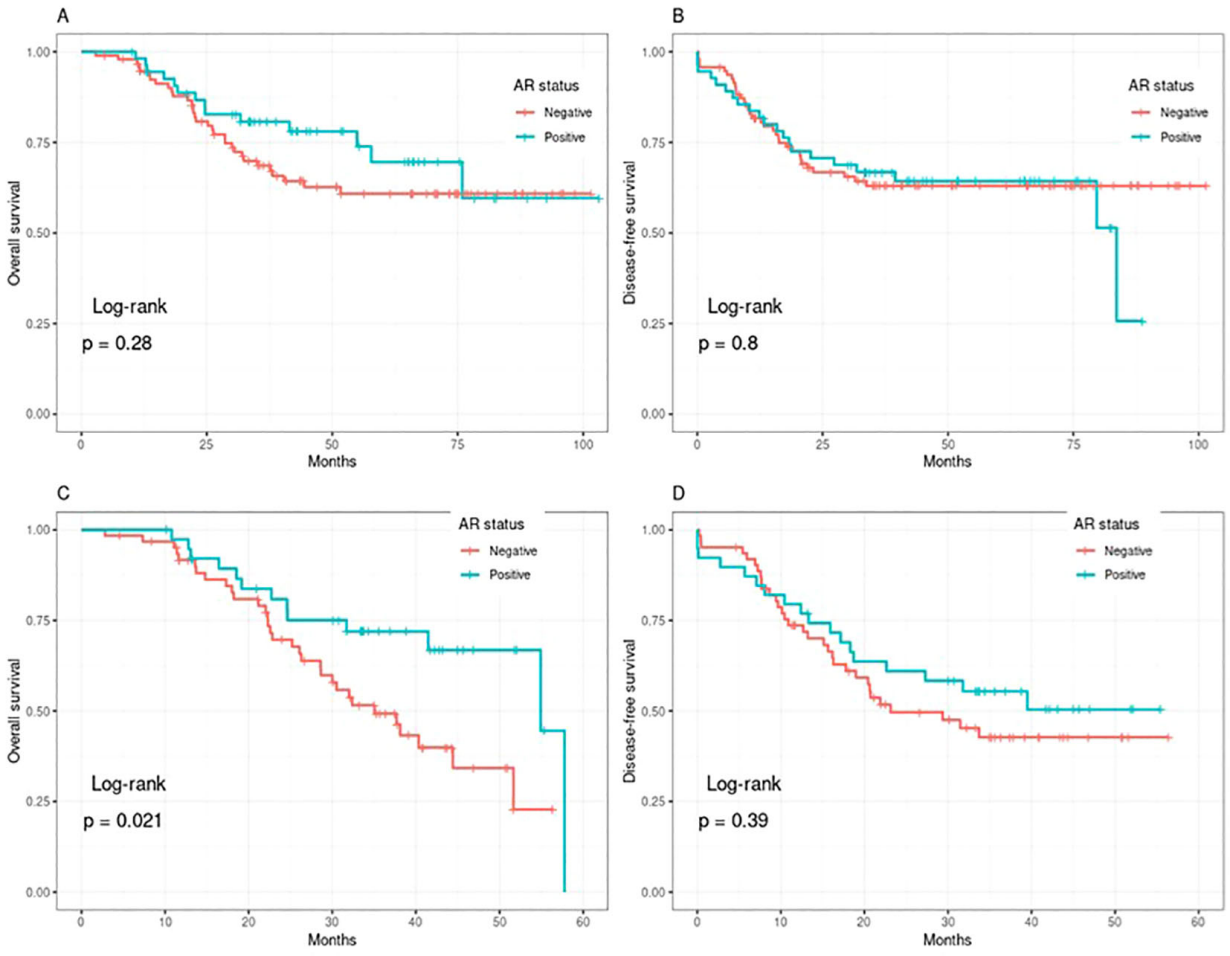
Kaplan–Meier survival curves comparing outcomes according to androgen receptor (AR) expression status. **(A, B)** depict overall survival (OS) and disease-free survival (DFS), respectively, while **(C, D)** illustrate 5-year OS and 5-year DFS.

The estimated 5-year DFS rates were similar for both AR-positive and AR-negative groups at 64.3% and 62.9%, respectively (*p* = 0.39) (**Figure 1d**). This similarity persisted with longer follow-up (*p* = 0.8) (**Figure 1b**).

The 5-year OS rates for the AR-negative and AR-positive groups were 60% and 70%, respectively, with a significant *p*-value of 0.021 (**Figure 1c**). However, this statistical significance was lost with longer follow-up (103 months), (*p* = 0.28) (**Figure 1a**).

**Figure 2:**
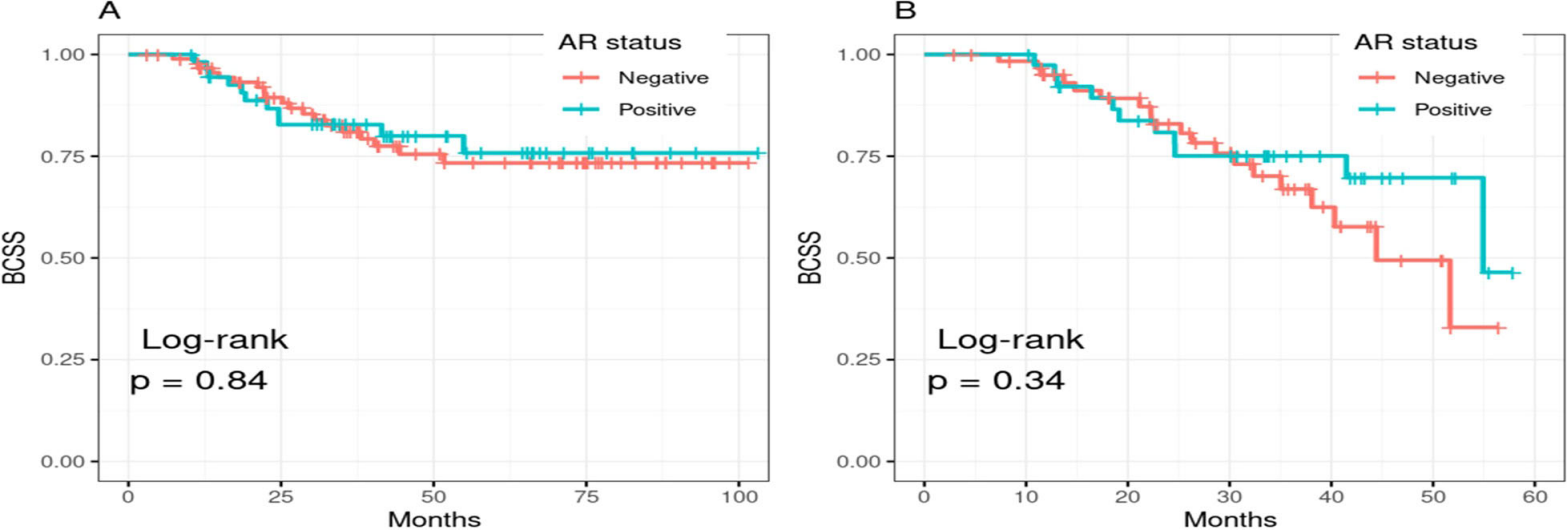
Kaplan–Meier curves of breast cancer–specific survival stratified by AR expression. **(A)** shows overall breast cancer–specific survival, and **(B)** presents 5-year breast cancer–specific survival.

The BCCS rates were compared between AR-negative and AR-positive groups. The 5-year survival rates for AR-negative patients were 73% (95% CI: 63.4-84.9) and for AR-positive patients, the rate was 75.8% (95% CI: 63.5-90.4). Statistical analysis revealed no significant difference in survival between the AR expression groups (*p* = 0.84) (**Figure 2b**). Further extended analysis also showed no significant differences in survival outcomes between these groups beyond 5 years (*p* = 0.34) (**Figure 2a**).

### 3.4 Univariate survival analysis

Univariate analysis of OS, DFS and BCSS was conducted on AR expression, tumor size, lymph node status, neoadjuvant CTX, adjuvant CTX, radiotherapy treatment, and surgery type were evaluated.

AR expression did not significantly impact OS (HR = 0.71, *p* = 0.3) nor DFS (HR = 1.07, *p* = 0.8). Tumor size was a significant factor, with T3 & T4 tumors showing worse OS (HR = 8.79, *p* = 0.003), DFS (HR = 9.21, *p* = 0.003) and BCSS (HR = 11.75, *p* = 0.017), compared to T1 tumors. Lymph node status was also significant, with N1 and N2/N3 stages showing increased hazard ratios for both OS, DFS and BCSS (all p-values < 0.01). Neoadjuvant chemotherapy was associated with worse OS (HR = 2.52, *p* = 0.006), BCSS (HR = 3.49, p = 0.006), but not DFS (HR = 1.58, *p* = 0.11). Adjuvant chemotherapy and radiotherapy did not affect OS, DFS nor BCSS. Patients undergoing mastectomy ± reconstruction had worse OS (HR = 3.99, *p* < 0.001), DFS (HR = 2.61, *p* = 0.002), and BCSS (HR = 5.48, *p* = 0.001) compared to those undergoing breast-conserving surgery. (**Table 2**).

**Table 2:** Univariate analysis of survival outcomes.

Parameters	Overall survival		Disease-free survival		Breast cancer specific survival	
	HR (95% CI)	P	HR (95% C)	P	HR (95% CI)	P
AR expression						
Negative	1		1		1	
Positive	0.71 (0.38-1.33)	0.3	1.07 (0.62-1.86)	0.8	0.93 (0.44-1.95)	0.839
Tumor Size						
T1	1		1		1	
T2	2.92 (0.68, 12.5)	0.15	3.9 (0.92, 16.5)	0.065	3.67 (0.48-28.22)	0.212
T3&T4	8.79 (2.08-37.2)	0.003	9.21 (2.17-39)	0.003	11.75 (1.56-88.54)	0.017
Lymph node status						
N0	1		1		1	
N1	3.39 (1.67, 6.87)	< 0.001	2.46 (1.33, 4.54)	0.004	4.56 (1.82-11.43)	0.001
N2&N3	5.07 (2.09-12.3)	< 0.001	3.91 (1.72, 8.92)	0.001	5.12 (1.56-16.84)	0.007
Neoadjuvant CTx						
No	1		1		1	
Yes	2.52 (1.3, 4.89)	0.006	1.58 (0.9, 2.79)	0.11	3.49 (1.42-8.57)	0.006
Adjuvant CTx						
No	1		1		1	
Yes	0.74 (0.42, 1.33)	0.3	0.9 (0.52, 1.53)	0.7	0.82 (0.40-1.68)	0.588
Radiotherapy treatment						
No	1		1		1	
Yes	1.04 (0.54, 1.97)	0.9	0.87 (0.49, 1.55)	0.6	0.93 (0.43-2.04)	0.864
Surgery type						
BCS	1		1		1	
Mastectomy ± Reconstruction	3.99 (1.98, 8.07)	< 0.001	2.61 (1.43-4.74)	0.002	5.48 (2.09-14.34)	0.001

HR, Hazard ratio; CI, Confidence interval.

### 3.5 Multivariable survival analysis

In the multivariable analysis of OS, DFS and BCSS, several factors that were significantly associated with survival in the univariable analysis were evaluated. AR expression was not significantly associated with neither OS (HR = 0.65, *p* = 0.2), DFS (HR = 1.00, p > 0.9) nor BCSS (HR = 0.84, p = 0.67). Tumor size had no significant impact on OS (T2: HR = 2.24, *p* = 0.3; T3/T4: HR = 4.23, *p* = 0.06) nor BCSS (T2: HR = 2.86, *p* = 0.322; T3/T4: HR = 5.14, *p* = 0.124), though larger sizes (T3/T4) were associated with worse DFS (HR = 6.35, *p* = 0.015).

Lymph node status was a significant predictor of both OS (N1: HR = 2.46, *p* = 0.017; N2/N3: HR = 2.94, *p* = 0.023) and DFS (N1: HR = 2.08, p = 0.026; N2/N3: HR = 2.80, *p* = 0.02). BCSS was significantly affected by N1 disease (p = 0.02) but not by N2/N3 disease (p = 0.107) compared to N0. Neoadjuvant chemotherapy did not show significant effects on OS, DFS nor BCSS, (*p* = 0.4, *p* > 0.9, and *p* = 0.123, respectively). Mastectomy +/- reconstruction was associated with worse OS (HR = 2.46, *p* = 0.022) and BCSS (HR = 2.98, *p* = 0.041) compared to breast conserving surgery, but surgery type had no effect on DFS (HR = 1.64, 95% CI: 0.85-3.16, *p* = 0.14). (**Table 3**).

**Table 3:** Multivariable analysis of survival outcomes.

Parameters	Overall survival		Disease-free survival		Breast cancer specific survival	
	HR (95% CI)	P	HR (95% CI)	P	HR (95% CI)	P
AR expression						
Negative	1		1		1	
Positive	0.66 (0.35, 1.28)	0.2	1 (0.57, 1.73)	> 0.9	0.97 (0.46-2.07)	> 0.9
Tumor Size						
T1	1		1		1	
T2	2.06 (0.47,8.99)	0.3	3.39 (0.79, 14.46)	0.1	2.66 (0.34-20.66)	0.4
T3&T4	4.18 (0.94, 18.51)	0.06	6.23 (1.4, 27.66)	0.02	5.11 (0.65-40.1)	0.12
Lymph node status						
N0	1		1		1	
N1	2.40 (1.14, 5.03)	0.02	2.06 (1.08, 3.9)	0.03	3.14 (1.2-8.12)	0.02
N2&N3	3.23 (1.31, 7.96)	0.01	2.64 (1.13, 6.17)	0.03	3.01 (0.9-10.07)	0.07
Neoadjuvant CTx						
No	1		1		1	
Yes	1.1 (0.53, 2.27)	0.8	0.86 (0.46, 1.6)	0.64	1.45 (0.56-3.76)	0.5
Surgery type						
BCS	1		1		1	
Mastectomy ± Reconstruction	2.47 (1.17, 5.22)	0.017	1.71 (0.89, 3.28)	0.11	3.14 (1.14-8.62)	0.03

HR, Hazard ratio; CI, Confidence interval.

## 4 Discussion

AR belong to the nuclear steroid hormone receptors family, which also includes Estrogen and Progesterone hormone receptors which are critical components of signaling pathways and play roles as transcription factors in regulation of gene expression. However, the biological role of AR is still uncertain. AR is expressed in normal breast epithelial cells and in 60-80% of breast cancer regardless of the ER status (17, 25, 32). Among TNBC, 10-35% of cases expressed AR (8, 25, 33). It was suggested that AR could be an emerging therapeutic target in breast cancer, especially TNBC (34, 35).

There is a disagreement on the prognostic significance of AR in TNBC. In a meta-analysis of 22 studies including 10004 patients, the subgroup analysis of TNBC revealed that AR expression significantly improved DFS and OS (HR 0.64, 95% CI 0.51-0.68, p-value <0.001 and hazard ratio 0.64, 95% CI 0.49-0.88, p-value <0.001 respectively) (14). In another meta-analysis by Kim et al. that included 16 studies, five studies with TNBC molecular subtype and AR expression were associated with significantly better DFS and OS (15). Other studies have suggested that AR expression could be an adverse prognostic marker in TNBC. A study with 94 stage III TNBC patients and a 23% AR expression rate showed worse DFS and OS in AR positive patients (25% versus 63%), although this trend was not statistically significant (25). Another study of 1467 patients by Hu et al. with a median follow up of 14 years showed TNBC patients with AR positive tumors had an 83% increase in overall mortality compared to those with AR negative tumors (HR 1.83, 95% CI 1.11-3.01, p = 0.02) (36).

In a meta-analysis of 27 studies including 4914 patients that investigated AR expression and survival outcomes in TNBC patients, univariate and multivariate analysis showed that AR expression had no statistically significant association with DFS nor OS, and concluded that in patients with TNBC, AR expression is not associated with prognosis regardless of confounding factors or heterogenicity of included studies (37). In this study, we did not find a statistically significant difference in clinicopathological characteristics of the patients and treatment modalities between AR positive and AR negative groups. This is in contrary to other reported studies were they showed an association between AR positive tumors and old age, low grade, larger tumor sizes and axillary lymph node involvement (38, 39). One possible explanation for this discrepancy could be differences in sample size, patient population, and methodologies, such as variations in AR detection methods, cutoff values for AR positivity, or clinicopathological factors not accounted for in this study.

We also did not find a statistical difference between AR positive and AR negative groups in relation to 5-year DFS (62.9% and 64.3%, respectively, *p* = 0.39). On the other hand, the 5-year OS was in favor of the AR positive group. (70% and 60%, respectively, *p* = 0.021). However, with extended follow-up, this difference disappeared with similar overall survival in the two groups. The observed late convergence in survival curves may reflect biological similarities between AR-expressing TNBC and hormonally responsive breast cancers, as steroid hormone receptor signaling pathways share downstream regulatory mechanisms that may influence tumor progression dynamics and long-term survival behavior. This pattern suggests that AR expression alone may not confer sustained prognostic advantage but may be associated with temporal survival effects rather than persistent outcome differences (40, 41).

In univariate and multivariate analyses, we showed that AR expression in TNBC is not a prognostic factor for survival, however tumor size, axillary lymph node status, and administration of chemotherapy remain prognostic factors. Patients who had mastectomy in our cohort demonstrated poorer DFS and OS compared to patients who had breast conserving surgery. This is in line with other studies which demonstrated that bigger surgery will not overcome bad biology (42–45).

Our study has several limitations. First, AR expression was assessed by immunohistochemistry using the institutional ≥1% cutoff; molecular profiling and alternative thresholds (e.g., 10%) were not available in this retrospective dataset. While molecular profiling may more accurately identify the luminal androgen receptor (LAR) subtype, it is not routinely used in clinical practice and remains cost-prohibitive (46). Second, the relatively small sample size may limit the statistical power of the analysis. Finally, although multivariable models adjusted for tumor size and nodal status, residual confounding related to clinical selection factors—particularly regarding surgery type—cannot be entirely excluded. Future studies are recommended to perform *a priori* power analysis to determine the optimal sample size required to detect clinically meaningful differences in survival outcomes between AR-positive and AR-negative TNBC patients. Larger multi-center studies with predefined statistical power calculations are encouraged to improve generalizability and confirm prognostic associations.

Our study is the first to describe this subtype of TNBC in the Levant region. Our study also had similar clinicopathological characteristic between the two groups and had an adequate follow-up duration.

## 5 Conclusion

In our cohort, androgen receptor is not a prognostic marker for patients with triple negative breast cancer. Several other prognostic factors exist and can be used to assess patients with TNBC. Considering the aggressive nature of and unfavorable outcomes of TNBC, and the scarcity of targeted therapy, the presence of AR expression in TNBC could be a potential target for antiandrogen therapy. We acknowledge that further studies exploring the therapeutic role of AR modulation, in TNBC, including both AR inhibitors and other therapeutic approaches, are warranted with larger, multi-center studies to further validate and generalize our findings.

## Data Availability

The raw data supporting the conclusions of this article will be made available by the authors, without undue reservation.
