# Supplementary material for: Assessing the prognostic role of androgen receptor expression in non-metastatic triple-negative breast cancer
Source: Front Oncol. 2026 Mar 23;16:1785283. doi: 10.3389/fonc.2026.1785283 (PMC13050708; doi:10.3389/fonc.2026.1785283)
Supplement: Supplementary file 1 [file Table1.docx]

Supplementary Material

# Supplementary and Tables

Supplementary Table S1. Kaplan–Meier Overall Survival Estimates by AR Status

| **Time (months)** | **Group** | **No. at Risk** | **Events** | **Survival** | **Std. Error** | **95% CI Lower** | **95% CI Upper** |
| --- | --- | --- | --- | --- | --- | --- | --- |
| 12 | AR Negative | 84 | 5 | 0.946 | 0.024 | 0.901 | 0.993 |
| 24 | AR Negative | 68 | 12 | 0.807 | 0.042 | 0.729 | 0.894 |
| 36 | AR Negative | 51 | 10 | 0.686 | 0.050 | 0.594 | 0.792 |
| 60 | AR Negative | 32 | 5 | 0.608 | 0.055 | 0.509 | 0.727 |
| 12 | AR Positive | 53 | 1 | 0.981 | 0.018 | 0.946 | 1.000 |
| 24 | AR Positive | 44 | 6 | 0.867 | 0.047 | 0.780 | 0.964 |
| 36 | AR Positive | 32 | 3 | 0.807 | 0.055 | 0.706 | 0.922 |
| 60 | AR Positive | 16 | 3 | 0.695 | 0.077 | 0.559 | 0.865 |
